# AAV‐mediated gene therapy as a strategy to fight obesity and metabolic diseases

**DOI:** 10.15252/emmm.201809431

**Published:** 2018-07-18

**Authors:** Carlos Henrique Sponton, Shingo Kajimura

**Affiliations:** ^1^ UCSF Diabetes Center San Francisco CA USA; ^2^ Eli and Edythe Broad Center of Regeneration Medicine and Stem Cell Research San Francisco CA USA; ^3^ Department of Cell and Tissue Biology University of California San Francisco CA USA

**Keywords:** Genetics, Gene Therapy & Genetic Disease, Metabolism

## Abstract

The fibroblast growth factor 21 (FGF21) is a member of the FGF superfamily that now comprises 22 members identified in humans. Unlike the canonical role of most FGF family members, FGF21 is released into the circulation and acts as an endocrine hormone by binding with low affinity to FGF receptors (FGFRs) as well as to the co‐receptor β‐klotho in the target cells to trigger the ERK1/2 and MAPKs signaling pathways. Described initially as a hepatokine, subsequent studies identified significant amounts of FGF21 transcript in the pancreas, the adipose tissue, and the skeletal muscle. FGF21 expression is highly regulated by environmental stimuli such as starvation, ketogenic diet, cold exposure, and exercise.

Our understanding of the physiological role of FGF21 and its pharmacological effects has expanded substantially over the last decade (Degirolamo *et al*, 2016). FGF21 exerts a multitude of metabolic benefits, including enhanced liver fatty acid oxidation, inhibition of gluconeogenesis, increased adipose thermogenesis, decreased inflammation in pancreatic β‐cells, increased glucose uptake in adipocyte/skeletal muscle, and leading to whole‐body improvements in energy homeostasis. Such improvements in metabolic parameters rapidly attracted the attention of biopharmaceutical companies to explore the therapeutic potential of FGF21 in metabolic disorders. Shortly thereafter, however, several disadvantages of utilizing native FGF21 peptide were identified, such as short half‐life and biophysical deficiencies (Kharitonenkov & Adams, 2013). In response, numerous analogs and mimetics of FGF21 were developed to overcoming the limitations of the native protein while maintaining the metabolic benefit. Such a new class of molecules includes optimized codon sequence of FGF21 (LY2405319—Lily Research Laboratories), fusion of Fc globulin fragment (Fc‐FGF21—Amgen), inclusion of non‐natural amino acid p‐acetylphenylalanine into the FGF21 for the site‐specific attachment of polyethylene glycol (PEG‐FGF21—Amgen), and agonist antibody to bind the FGFR–β‐klotho complex (mimAb1—Amgen) among others (Kharitonenkov & Adams, 2013; Degirolamo *et al*, 2016). These variants of FGF21 proved to be effective in improving metabolic health in animal models and clinical trials. For example, the variant LY2405319 demonstrates effectiveness in decreasing body weight, improving lipid metabolism (e.g., decreased total cholesterol, triglycerides, LDL, and increased HDL), and enhanced insulin sensitivity, despite modest effects on fasting glucose levels in two PHASE I clinical trials (Kharitonenkov & Adams, 2013; Degirolamo *et al*, 2016). Despite the pharmacokinetic improvements, FGF21 analogs/mimetics require routine administrations, which may compromise patient adherence and raise immunological concerns related to the use of exogenous proteins (Degirolamo *et al*, 2016).

In this issue, Jimenez *et al* (2018) took advantage of adeno‐associated virus (AAV) and its ability to mediate a long‐term, sustained protein production *in vivo*, such that it circumvents the major limitation of FGF21 analogs/mimetics. AAV vectors, derived from non‐pathogenic viruses, are predominantly non‐integrative vectors that persist for years as episomes in the nucleus of non‐dividing cells (Lisowski *et al*, 2015). Multiple studies in animal models and clinical trials have reported the AAV therapeutic efficacy with minimum clinically significant adverse events (Lisowski *et al*, 2015). The authors demonstrated that one single administration of AAV‐FGF21 under control of a liver‐specific promoter (hAAT) resulted in sustained levels of circulating FGF21 up to 1 year, which has been the most extended follow‐up FGF21 study reported in animal models so far.

The administration of AAV‐FGF21 in diet or genetic obesity mice models resulted in significant metabolic benefits similar to previous findings (Degirolamo *et al*, 2016). Jimenez *et al* (2018) reported reductions in body weight, adipocyte size, and inflammation, as well as reduced hepatic steatosis and fibrosis (Fig 1). The improvements in energy expenditure and glucose homeostasis were linked to increased non‐shivering thermogenesis, glucose uptake in brown or white adipocytes, normalization of adiponectin and leptin levels, as well as decreased pancreatic islet size with consequent reduced fasting insulin and glucagon levels. Interestingly, no change in hepatic gluconeogenesis was observed which is contrary to previous studies (Degirolamo *et al*, 2016). Altogether, these findings reinforce the therapeutic efficacy of AAV‐mediated FGF21 gene therapy, resembling many of the metabolic outcomes already reported by using pharmacological FGF21 analogs/mimetics.

**Figure 1:**
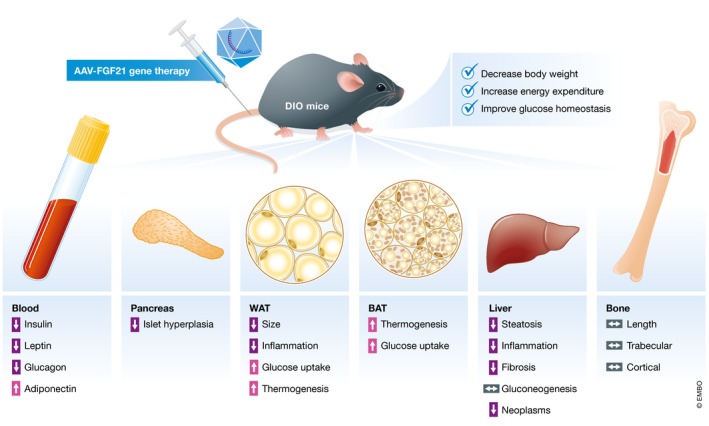
AAV‐FGF21 gene therapy improves metabolic health in diet‐induced obesity mice (DIO) Jimenez and colleagues demonstrated that adeno‐associated virus (AAV)‐mediated FGF21 expression promotes the following metabolic benefits in indicated tissues.

While the metabolic benefits of FGF21 treatment have been unquestionable, the underlying mechanisms by which FGF21 improves metabolism have been a matter of debate. For instance, the present study demonstrated increased expression of uncoupling protein 1 (UCP1) and reduction in lipid content in brown adipose tissue (BAT), indicating increased thermogenic activity in the adipose tissue—this is also supported by others (Owen *et al*, 2014). On the other hand, several studies showed that FGF21 increased whole‐body energy expenditure even in mice with ablated interscapular BAT or UCP1 knockout mice, suggesting an UCP1‐independent mechanism in response to FGF21 therapy (Emanuelli *et al*, 2014; Samms *et al*, 2015). Curiously, the present study found that AAV‐mediated FGF21 increased the expression of phosphatase orphan 1, an enzyme involved in the creatine‐driven substrate cycle (Kazak *et al*, 2015), and ryanodine receptor 2, a key component in the Ca^2+^‐cycling thermogenesis in the adipose tissue (Ikeda *et al*, 2017), both of which control UCP1‐independent thermogenesis. Because FGFRs are known to modulate intracellular calcium levels in response to FGFs, these new data may suggest a contribution of UCP1‐independent thermogenic mechanism in response to FGF21 gene therapy. Of note, a recent study showed by using adipocyte‐specific β‐klotho (*Klb*) receptor knockout mice that BAT mediates the glucose‐lowering effect of FGF21, whereas the anti‐obesity effect of FGF21 appears to be through non‐adipose tissues (Bon Durant *et al*, 2017). Thus, future studies are necessary to unveil the molecular and cellular mechanisms by which FGF21 treatment improves metabolic health.

It should be noted that some concerns have been raised regarding the effects related to FGF21 therapy. For example, FGF21 administration can decrease bone mass or promote torpor, a condition where core‐body temperature and physical activity are reduced (Kharitonenkov & Adams, 2013). However, the AAV‐mediated FGF21 gene therapy increased energy expenditure and physical movement with no signs of trabecular or cortical bone loss or changes in bone length. Given the growth factor nature of FGF21 family, the potential tumorigenesis in long‐term therapies is also a worthy factor to be addressed. Nonetheless, the present study found that AAV8‐hAAT‐FGF21 was protected from obesity‐associated liver neoplasms, which in turn argues in favor of a protective role of FGF21 against malignancies in the liver. Finally, the authors demonstrated that alternative tissues, such as adipose tissue and skeletal muscle, can be used as host tissues to overexpress FGF21 in cases of liver diseases, including cirrhosis or liver cancer.

In conclusion, Jimenez and colleagues provided a new and efficient way to induce long‐term high‐circulating FGF21 levels *in vivo*. The AAV‐mediated increase in FGF21 recapitulates most of the previously reported metabolic benefits of pharmacological FGF21 analogs/mimetics while avoiding essential obstacles such as treatment compliance and immunogenic reactions. The present study opens a new possibility that the AAV‐mediated gene therapy can be applied to fight obesity and metabolic diseases.
